# Getting it right! Enhancing youth involvement in mental health research

**DOI:** 10.1111/hex.12386

**Published:** 2015-07-22

**Authors:** Lauren Mawn, Patrick Welsh, Lauren Kirkpatrick, Lisa A.D. Webster, Helen J. Stain

**Affiliations:** ^1^ Mental Health Research GroupSchool of Medicine, Pharmacy & HealthDurham UniversityStockton on TeesUK; ^2^ Tees, Esk and Wear Valley NHS Foundation TrustStockton on TeesUK; ^3^ Youth Speak on Mental Health ResearchDurham UniversityStockton on TeesUK

**Keywords:** adolescent, involvement in research, Patient and Public Involvement, youth

## Abstract

**Background:**

Few studies relating to youth mental health have actively involved young people in the design and conduct of research.

**Aims:**

This qualitative study explores the perceptions of young people about involving them in mental health research.

**Method:**

An opportunistic sample of eight young people (aged 14–24 years) from non‐statutory mental health organizations was interviewed. Interviews were transcribed verbatim, and inductive thematic analysis was conducted.

**Results:**

Six key themes emerged reflecting a desire for young people to have the opportunity to actively contribute to every stage of the research process. Meaningful research involvement was perceived as offering opportunities to develop personal skills, contribute to making a difference and ensuring research projects were more relevant.

**Conclusions:**

Young people with an active interest in mental health promotion demonstrate a desire to be involved in research with training in research methods likely to facilitate this process. Researchers need training on how best to actively and meaningfully involve young people in mental health research.

## Introduction

In the United Kingdom (UK), Patient and Public Involvement (PPI) in the development and execution of mental health research and service reform has become an essential element.^1^, ^2^ The term PPI has been introduced to encourage involvement of service users and carers in health services, which was then adopted for research. However, there exists little examination of the implementation of involvement in youth mental health research. This article seeks to explore involvement of young people in mental health research. For the purpose of this study, PPI is conceptualized as any involvement stemming from one off consultation to co‐investigator roles across the various elements of research (e.g. from advising on recruitment to being involved in the conceptualization of a study and writing the grant application or paper). Evidence suggests that studies involving patients and members of the public are more robust, recruit to target and enhance the translation of findings into practice.^3^ In health research, young people have been excluded from the process of shaping research, and when they are included their perspectives are often filtered through the interpretations of adult researchers.^4^

Within mental health research, the lack of involvement of young people in the design, development and implementation of robust, high‐quality research is evident by the paucity of published research articles acknowledging and describing meaningful involvement. Indeed, a review of studies engaging PPI approaches across the field of health and social care research found few projects focussing upon youth mental health.^5^ Of those studies that were identified, the majority failed to involve young people themselves but obtained input from carers and teachers instead.^6^, ^7^, ^8^ Interviews with principal investigators working on UK Clinical Research Network Portfolio (http://public.ukcrn.org.uk), covering research topics affecting those across the lifespan, note that there is often a lack of understanding from academic researchers about how to involve patients and members of the public effectively, resulting in a poor experience for both parties.^9^ Although several guides exist relating to the involvement and engagement of young people in research,^4^, ^10^, ^11^, ^12^ none have specifically focused on understanding and exploring these processes within the context of youth mental health. By listening to the perspectives of young people, we can identify barriers to involvement to date and promote future positive practices.

The aim of this study was to explore the perspectives of young people with an existing interest in mental health promotion regarding: (i) how best to involve young people in mental health research execution and design; (ii) some of the barriers and challenges of involving young people with mental health difficulties; and (iii) how to reimburse young people for their contributions.

## Method

### Participants

Young people aged between 14 and 24 years were recruited opportunistically from three mental health charities and organizations based in the north‐east of England (*YoungMinds* north‐east, *Change UR Mind* and *Youth Speak*). This process was adopted to include young people who had prior knowledge, experience and/or interest in mental health difficulties. It was deemed that these young people would be ideally placed to comment upon factors and barriers which may be unique to mental health research and practice.

### Research design

Semi‐structured interviews were conducted at a time and venue convenient to the young person. This approach was chosen as it allows the young person (the experiential expert) the opportunity to lead topic discussions whilst offering the interviewer flexibility to probe interesting areas that arise and explain concepts in a variety of forms.^13^ Participants were invited to attend the interview with a friend, a family member or were given the option of pairing with another participant in order to reduce possible interview anxiety. The content and structure of the interview schedule was informed by the published literature, in consideration with the aims and objectives of the study. To ensure comprehension and relevance to young people, the interview schedule was piloted with a young person involved in the study (LK). Thus, following a section of introductory questions, participants were guided to explore ideas in relation to specific stages of the research process (e.g. idea generation, research design, data collection, data analysis, dissemination). All interviews were conducted between January and March 2014 and lasted between 25 and 40 min (Please see Appendix 1 for a copy of the interview schedule). As analysis of the interview transcripts occurred concurrently with data collection, and sample size was determined by theoretical saturation, that is, recruitment and data collection continued until additional interviews added no new meaningful data.

### Data analysis

All interviews were recorded using digital audio equipment and then transcribed verbatim. Transcripts were analysed using a thematic approach guided by the processes outlined by Braun and Clark.^14^ In the first instance, each transcript was analysed separately through a process of re‐reading, descriptive coding followed by a more interrogative examination of the transcript at a higher conceptual level. Key ideas, words and quotations were highlighted and formed the basic units for analysis. Once each transcript had been analysed, patterns and connections across transcripts were identified by a process of abstraction. All emergent themes were placed in a list and then reviewed with the most frequent or potent themes being moved to form clusters of related or super‐ordinate themes.^13^ Themes were therefore generated by an inductive approach, avoiding prior assumptions and hypotheses. Initial analysis of all transcripts was undertaken by PW. Generated themes revised and agreed upon through a series of consensus meetings with a separate analyst (LM), who analysed the data independently prior to meeting. To enhance scientific rigour, identified themes were presented and discussed during a focus group attended by three study participants. The focus group allowed participants the opportunity to question and amend the name and content of super‐ordinate themes and select supporting quotations. This process ultimately ensured that findings reflected the participant's perspective and not the interpretations of the adult or professional researcher (a means of testimonial validity).^15^

### Ethical approval

The project was approved by the Durham University School of Medicine, Pharmacy and Health Ethics Committee. As participants were recruited from three mental health charities, NHS ethics was not required. Inclusion criteria for the study were (i) aged 14–24 years; (ii) previous or current experience of obtaining care from mental health services; and (iii) members of relevant charities and youth groups. Individuals were excluded if they were unable to demonstrate Gillick competence, which was assessed prior to consent and interview. Written informed consent was obtained from all participants prior to interview. All participants were given a £10 voucher for participation in both the interview and the follow‐up focus group. Travel expenses to and from the interview and focus group were also paid. All participants were randomly assigned initials during the study to ensure anonymity.

## Results

All individuals (*N* = 8; mean age = 18.1; standard deviation = 3.31; age range: 14–24 years) met inclusion criteria, provided consent and were interviewed for the study. Three of these young people participated in the focus group discussion. The majority of participants were female (*n* = 7). From the data analysis, six key themes emerged (See Table 1 for a summary) and were subsequently named by focus group participants.

**Table 1:** Summary of identified themes

Theme	Summary
Research‐What does it mean?	Preconceptions which can generate feelings of anxiety
The Research Roundabout (Cycle)	Involvement in all stages – involve young people early in this process, ensures motivation, interest and creativity
	Data analysis – Boring, difficult, requiring lots of training. Opportunity to develop skills if supported
	Sharing research findings – Opportunity to be creative and showcasing success
Giving Back	Personal development – Chance for young people to develop new skills and change people's lives
	Reimbursement – Refreshments and travel expenses
	Incentives – Other factors more important than vouchers and payment
Getting young people through the door	Events and festivals – to generate interest
	Organizations and schools – Approach youth organizations, involve young people to share their experiences of research and involvement
Barriers	Anxiety – Taking time to explain things, offering advice and guidance
	Understanding mental health – Ensuring young people are able to cope with the demands of involvement
	Stigma – Fear of stigma because of involvement
	Life – Recognition that young people have busy lives
	Formality – Prevents involvement and reduces productivity
Technology and face‐to‐face communication	Both approaches should be utilized where possible to maximize contributions from a range of young people

### Research – what does it mean?

Participants defined the term ‘research’ as ‘looking for new information’ (AA) or ‘just generally to find a new way of doing things’ (BB). However, participants had preconceived ideas when discussing research involvement within the context of mental health:

Research often meant sort of trialling like medication and things like that…So you've got that preconceived idea that you're going to be asked to take some sort of medication (CC)

This perception generated feelings of anxiety for some young people and arose due to the assumption that a high degree of intelligence was a necessary pre‐requisite for research involvement:

When I thought of research at first it was like thinking I've got to be brainy to be able to do it, I've got to have had a PhD, do you know what I mean…I don't think enough young people know what research actually entails, because it's like well actually it's very different to my first impressions (GG)

### The research roundabout (cycle)

The following theme reflects an exploration of the research process or cycle with those interviewed stating that young people can and should be involved in all stages of research. However, it was extremely important that young people should be involved as soon as feasibly possible as this was perceived as enhancing motivation, interest and idea generation:

If you're a young person who's thought of the idea, you have ownership over the project, whereas if you come into a later stage you're not going to have as much ownership over that project and you might not feel as passionately about it. So I think it's really important for you to get the ideas to begin with and then have it as a process that you go with and travel with them [researchers] through that process, so that they learn something and you learn something from it (HH)

Researchers who fail to engage or update young people as the research progressed were criticized:

Yeah, well in a way I guess you've got to keep them [young people] updated with what's going on, because if they're involved in something and they're not really sure what's happening with it or where it's going or if it's even achieving anything, then they're going to be thinking is there any point doing this (FF)

Being involved in the data analysis stage of a research project was generally associated with boredom and there was a perception that it required a lot of time, training and expertize:

Probably a feeling of, ‘oh I won't be able to do that, that's too clever, too big, too’; it's that would be the initial barrier, I guess…I think it's probably not as active as the other stuff so you will just be sitting in an office with someone (HH)

However, data did emerge representing a deviant case^16^ whereby the same participant later reflected how the opportunity to conduct data analysis would develop their skills and enhance the analysis process:

For me it [data analysis] would be a good challenge and it would be something that, again if I wanted, if it was me I would want to go through the whole process from start to finish and obviously that's one of the main, most important bits and it would be good because as a young person we might have different perceptions so it could influence, like that you [researchers] might see one thing and we might see another in the data (HH)

Finally, involving young people in the dissemination stage was identified as an opportunity to challenge stigma, create an impact and celebrate partnership working:

I think things like that are always better off coming from the young person directly. I think it makes more of a statement within sort of the media and things. Because there's a lot of negativity surrounding young people at the minute that we don't do anything, we're quite lazy and, you know, I think it would make that statement of well actually no we're not lazy, this is what we found, you know, and we've been involved with it all the way through (CC)

### Giving back

To facilitate meaningful involvement, it was expected that adult or professional researchers would provide personal development opportunities and various expenses and incentives during research projects. One area identified was the opportunity to add experiences to curriculum vitaes (CVs) and educational and employment application forms:

People also think about what they can put on their CV or like their UCAS form and things, things like that. And it's just like character building…Yeah, building my skills up (FF)

Alongside the gains of learning new skills, the opportunity to help others and instigate change through research involvement was also highly valued:

You'll have the say on a big thing possibly that could help to change something. And by being part of that is like helping other young people. Because it won't just be you who'll have the same feelings, there'll be a lot of other young people and you're basically being a voice for them (BB)

In terms of recognizing the time and contribution of young people, providing reimbursements in the form of travel expenses and refreshments was important as this demonstrated respect and value for their contributions:

If it's going to be all day, then you can't expect them [young people] to have like, bring their own food and stuff like that, because it's like wrong in a way of saying ‘oh well we want you to do this but you've got to pay your own travel and you've got to bring your own food’, because they're not going to do it (BB)

Finally, payment or gift vouchers were deemed appropriate forms of reimbursement, although these were not always perceived as essential:

I think it's important because you don't want people to be out of pocket for helping. But it's like I think, I don't know. I think the gift vouchers are a great idea, but I don't think it's kind of vital…The most important thing for me, like I say, would be learning and making a difference (GG)

### Getting young people through the door

Several ideas were suggested in how best to recruit young people to be part of a research team. These included the use of leaflets, posters, oral presentations and information stalls at youth focused events and festivals:

I think it's about getting out there…even if it is going out to events and having a stand there. It can be exhausting if you're there all day, but I think it's one way of getting young people involved (GG)

Approaching existing mental health charities and organizations was perceived as a more appropriate starting point in order to facilitate youth involvement, before eventually moving onto schools and colleges in order to ensure a mix of young people and ideas:

The people that are part of [Charity X] are dedicated people that probably will come along, and you know they're interested already. They've already seen the opportunity of going into a group. But maybe there's just some people in schools [that]) haven't, don't know it's out there. So I think if you give other people a chance to come along…I think it's good to have a bit of both because then you've got some people with maybe experience and then other people that are interested and new and wanting to share their ideas (FF)

If researchers wanted to recruit young people from school settings, it was important that engaging workshops and assembly sessions were offered using example research tasks or taster exercises:

I think assemblies can be extremely [difficult], you have to really put on an amazing assembly to get any interest because I remember when I was at all the assemblies external speakers used to bore the hell out of me! And I didn't ever want to do the stuff that they were offering even though they might have been offering the most amazing thing, so if you were to go into schools, maybe workshops and stuff, giving them a taste of what you're going to be offering rather than just going and telling them what you're offering (HH)

In addition, these workshops should be facilitated in conjunction with young people working on the research project or with prior involvement experience:

Again I'd even try and put it from the young person's perspective of what you're trying to achieve…Often other young people will then become inspired and you think well they're like me, they're the same age, well maybe I could do that, you know (CC)

### Barriers

Participants recognized that feelings of anxiety could be a significant factor in preventing involvement. As previously discussed, anxiety could be experienced as soon as young people are approached to be involved. Researchers therefore should be mindful to build rapport prior to commencing research involvement:

I think it might be a good idea to kind of like thoroughly go through what would be like the process of it, and like so they could make sure that they really understand what's going on and what's gonna be asked of them (DD)

Lack of support from the research team and the fear of others taking credit for young people's contributions emerged as common anxieties that may be experienced prior to and during a project:

Just the time and if you don't get support it can be extremely hard on your stress levels and it's a lot harder to cope if you're having to do it yourself and things like that (HH)

Mental health was also identified as an important factor with the recognition that involvement could offer positive personal development opportunities and a chance to make a difference. However, involvement could trigger or escalate existing mental health difficulties for some young people:

I guess it depends on every different person. Everybody is going to have different issues and things, and you've just got to look at that…I think sometimes people who are unwell being involved in mental health, and then the research, that kind of thing, sometimes it might make them more unwell maybe by speaking about it and hearing about it (CC)

Generally, it was felt that young people needed to be in a position or state where they were able to cope with the demands of research involvement and deal with potentially sensitive or upsetting information. As such researchers have a responsibility to identify, discuss and minimize possible difficulties prior to involvement so that these issues can be managed with the young person:

I think, well if you think about, I don't know, somebody who has got depression, they might not be able to get out of bed, never mind coming along and doing an interview. I think mentally you've got to be in a place where you can think about doing it and be capable and stable to be able to do it, because the thing is there might be triggers, and it's being strong enough to be able to cope with that. You know, I mean it's like because things crop up all the time in life, and so some things might touch a nerve or whatever, and it is being strong enough and well enough to be able to do it (GG)

Stigma emerged as a barrier to involvement with recognition that some young people would not want to be associated with a research project that investigated mental health issues for fear that others may find out:

Some people don't want their friends to know; they don't want their parents to know; they don't want the rest of the family to know (CC)

Peer pressure maybe might stop people, because it's almost like ‘oh I might get involved in that’, but if they're just thinking their friends were like ‘oh that's really uncool’, then they might not want to anymore (EE)

General day‐to‐day life also emerged as a barrier to involvement, with young people busy managing a variety of competing interests including school, college, jobs and friends. The role of the researcher was therefore to understand and respect this. Researchers should also ensure meetings are arranged around other commitments and that any meetings represented ‘value for your time’ (HH). Some examples of this already exist, whereby Youth Speak meet during the evenings and the CRN Mental Health Young Persons Advisory Group meets on a Saturday.

A final barrier inhibiting involvement reflected the concept of formality in research meetings. For example, meetings had to be engaging, recognize possible power imbalances and allow for regular breaks to aid concentration and social interaction:

I think to begin with in the first couple of sessions [at Charity X] it wasn't as youth friendly as it is now…because we turned up and [Person X and Y] were standing at the front and talking to us…standing at the front in terms of like as if it was a lecture kind of thing…but having us round a table in a similar kind of level, I don't know whether that would work, sharing roles within the group as well so getting young people to write on the flipchart (HH)

### Technology and face‐to‐face communication

This theme focused on young people working together during a research project using online forums and face‐to‐face group working as important communication approaches. For example, online forums and social media could meet a need for some individuals who struggled with issues such as social confidence as well as allowing young people to contribute at any time during the day rather than being restricted by a set meeting time:

There are people especially those who've got mental health problems their self [who] won't want to leave the house…That's why like if it can be done online as much as possible you can get like the generic feeling and they can do it at any time (BB)

However, some participants noted that face‐to‐face meetings enhanced motivation, promoted opportunities to generate better ideas as well as meeting other people:

If you're by yourself you might not always notice things, or you might not be as motivated to do it. You might think of it more as work, extra things put on you, rather than working together and seeing it as a fun thing (FF)

A flexible approach could be undertaken that allowed individuals to attend face‐to‐face meetings in order to listen to ideas without the fear of speaking whilst later contributing their ideas and views online after the meeting:

I think personally it's better to come together [as a group], but then I like to talk, so I would do, but then you might get people who, like in the board meeting you get people that kind of don't say a great deal; however, their ideas are just as important. And so it could be good to have like either them write it down or email and contribute that way if they don't feel comfortable talking in the group, so then I think it is important to be able to adapt to the individual (GG)

## Discussion

Using interviews as an exploratory method, the paper describes the key themes of young peoples’ perceptions of PPI in mental health research. To our knowledge, this is the first time the views of young people have been collected on this topic using a research‐based approach. Whilst young people in this study felt they can and should be involved at all stages of a research project, previous surveys relating to PPI research practice in the UK^17^ and existing bibliographies of published research^5^ do not reflect a state of widespread youth involvement in mental health research. Within our population of participants, this lack of research involvement does not appear to be the result of ambivalence or poor motivation inherent in young people themselves but potentially an absence of opportunities. This needs to be investigated further but could stem from researcher anxiety in relation to perceived resource commitments in undertaking a project that offers PPI contributions that are both meaningful and beneficial to everyone involved.^4^, ^18^

The current analysis highlights that young people with a pre‐existing interest in mental health wish to contribute to research especially when projects offer personal development opportunities and a sense of impact by making a difference to others. These findings mirror personal accounts collected from adults involved in health research, who have described that having a ‘voice’ and bringing about change to service practice are the main factors for research involvement.^19^ Indeed, guidance from the National Children's Bureau promotes this ‘up‐skilling’ of young people through the provision of training opportunities and using certificates to recognize and document development.^11^ However, commentators have challenged this aspect of PPI suggesting that a lay person may not retain their ‘lay’ perspective or unique contribution when trained beyond certain standards.^20^, ^21^

The identification by our participants that research involvement should ideally take place at the conceptualization of a project is indeed supported elsewhere. Prior research regarding patient involvement in quality improvement projects in the NHS found that early involvement brought many perceived benefits including a clearer understanding of the project's aims and objectives and better team cohesion.^22^ In spite of this, there is no subsequent evidence to support that this is indeed common research practice. Considering the results of this study and evidence that involving patients and public results in more robust research,^3^ it is important for future research to examine *how* youth involvement enhances the robustness of research. For example, does involvement make information sheets better? Does involvement help to ensure that research question(s) are more relevant to the needs of young people?

Finally, young people identified several factors that may prevent PPI including anxiety, on‐going mental health problems, stigma and a busy lifestyle. Thus, researchers need to acknowledge these concerns by explaining tasks, understanding an individual's strengths and weaknesses, whilst identifying and managing the possible impact of any mental health difficulties. In guiding researchers in managing on‐going mental health problems, the Putting Participation into Practice guidelines published by *YoungMinds* 23 directs investigators to consider a young person's mental state and the provision of support prior to and during engagement activities. Similar to our own findings, they highlight that some young people may lack confidence and require a sustained period of time to build trust and rapport with professionals.

## Strengths and limitations

Although it is apparent that many of the themes and findings identified in this study overlap with guidelines already published, this study provides novel insight by capturing rich personal accounts from young people on their thoughts, ideas and prior experiences in relation to mental health research. Our recruitment strategy of interviewing young people who were members of specific mental health organizations and charities ensured that those interviewed were able to draw and reflect upon knowledge of mental health issues and engagement approaches experienced firsthand. However, it could be argued that this resulted in a sample of potentially highly motivated and engaged adolescents as participants. This combined with a predominately female cohort means that these findings may not be generalizable to other adolescent populations. Replication is therefore required in youth communities with little or no interest in mental health issues.

Another strength of the current study is that the definition of PPI was kept relatively broad, including any involvement stemming from one off consultation to co‐investigator roles. Our study did not lead young people to have a fixed view of involvement by providing a formal definition, as such there was no restriction on themes generated. Indeed, involving young people in the process of defining of PPI for mental health research may be an important avenue for future research, especially considering the diverse understanding of PPI both nationally and internationally. As a study investigating youth involvement, the study benefitted from the input of author LK as a co‐investigator/young person. On reflection, this involvement had the most significant impact in the process of recruitment, offering possible participants the chance to talk to another young person or peer about the project. However, a more inclusive research role for our co‐investigator could have been to act as a coder during data analysis and/or to interview participants. The use of peers with experiences of mental health services to conduct interviews has been utilized previously and demonstrated the elicitation of more critical information.^24^, ^25^

## Implications

Our findings support many of the published guidelines relating to youth involvement in research whilst also highlighting pertinent factors relevant to conducting mental health research. The themes identified also provide some guidance for researchers to involve young people in a constructive, non‐piecemeal way that is ultimately beneficial to all parties (i.e. supporting and training young people to develop workshops and presentations in order to recruit other young people). The motivation and desire of those interviewed is a positive indication for the future of PPI given that young people wish to contribute to mental health research in spite of possible stigma, peer pressure, personal difficulties or multiple life commitments. Although speculative, the need to be flexible in communicating, arranging meetings, offering development opportunities and suitable reimbursement may collectively be more important in sustaining interest and engagement within this age group, as in our experience young people may be quicker to disengage and less likely to challenge authority in comparison with adults with an interest in PPI. Ensuring young people have a positive experience of research involvement is also important as these individuals may continue to engage or participate in research for many years or indeed pursue a future career in research or practice. Therefore, researchers and clinicians may benefit from youth cultural training to facilitate non‐piecemeal involvement of young people in their future research.

Currently, it is unclear how many young people have actively contributed to the design and execution of mental health research projects within the U.K. Although we are aware of two youth mental health research groups that facilitate PPI (the NIHR CRN: Mental Health Young Persons Advisory Group and Youth Speak at Durham University),^26^, ^27^, ^28^ it is difficult to explicitly identify youth‐related contributions to research even when projects have been published as peer‐reviewed articles. A national mapping survey similar to that conducted by Patterson *et al*.^17^ is therefore warranted in order to fully understand current practice. Secondly, our research does not address the practicalities of managing available resources and other research processes when young people are involved via PPI throughout the entire lifecycle of a research project (e.g. ensuring confidentiality, managing disagreement, conducting risk assessments). Further investigation into the possible barriers that prevent youth involvement in mental health research, from the perspective of researchers, commissioners of services and funding bodies would therefore be a valuable addition to an understudied area.

## Ethical approval

The project was approved by the Durham University School of Medicine, Pharmacy and Health Ethics Committee. Written informed consent was obtained from all participants prior to interview. All participants were given a £10 voucher for participation in both the interview and the follow‐up focus group. Travel expenses to and from the interview and focus group were also paid. All participants were randomly assigned initials during the study to ensure anonymity.

## Funding

The study was supported by a small grant from the Mental Health Research Network (now NIHR Clinical Research Network: Mental Health).

## Declaration of interest

None.

## Appendix 1 Interview schedule

### Welcome

Thank you for agreeing to take part in our research project.

As you will have read in the information sheet, the aim of this interview is to discuss your ideas of how to engage and involve young people in mental health research.

I am here to guide the discussion, and I have a set of pre‐prepared questions. However, I want you to remember that

- there are no right or wrong answers,
- please speak freely we want as many ideas as possible,
- all your opinions are important, and
- do not worry about being on the right track, we want to hear your views not our own.

We are recording the session so we do not miss any of your comments. Although quotations will be used within the final report from individuals, the information you provide will be confidential. Quotations, therefore, will not be attributed to you as an individual and will be anonymous.

Do you have any questions before we begin?

1. Firstly as a bit of a background can you tell me a bit about how you became involved with Change UR Mind, Youth Speak or YoungMinds?

(Prompts: Duration, Specific projects you have worked on)

This project is about engaging and involving young people in mental health research. When we talk about research, we often mean testing things to find out new information and to improve things for other people. For example, research might be asking 100 people to fill in a questionnaire every week about how happy they are feeling and looking to see whether some people are happier than others, or asking people with anxiety to take a new drug for 6 weeks to see whether it makes them better and monitoring their symptoms.

Although I have told you what I think research means, how would you have described the word ‘research’ if I hadn't given this description and example?

Research is often seen or described as a process (see flowchart) in terms of thinking up ideas, agreeing on an idea, planning the research/study, then collecting information or data, looking at the results and then finally sharing the findings.

Looking at this flowchart which areas do you think YP could be most involved and why?
If we break the research process into these stages. Could you tell me a bit more about how you think young people could be involved at stage 1, 2, 3, 4, etc? What could that involvement look like, what kind of things could young people do here?
What things would make you want to be involved?
As researchers we are keen that young people get something back for being involved in research and giving up their time. Do you think young people should be reimbursed for taking part in research?
Often it is difficult to get young people involved in research, how do you think researchers could find young people to get involved in research? Who should these young people be?
What kind of issues might stop a young person from being involved in research?
How do you think mental health issues may affect young people being involved in research (if at all)?
Is there anything else that we have not covered that you would like to add in relation to young people being actively involved in the research process? Have you experienced anything that has worked well?
